# *Exposure to copper metal enhances the tolerance of An. gambiae s.s. over multiple* *generations while reducing both fertility and fecundity in this primary malaria vector* *.*

**DOI:** 10.12688/wellcomeopenres.23229.2

**Published:** 2025-02-05

**Authors:** Massioudou Koto Yérima Gounou Boukari, Genevieve Tchigossou, Innocent Djègbè, Ghislain T. Tepa-Yotto, Eric Tossou, Donald Hessou-Djossou, Camille Dossou, Louckman Monra Seidou, Aldo Emmanuel C. Glokpon, Danahé Adanzounon, Adam Gbankoto, Rousseau Djouaka

**Affiliations:** 1 Département des Sciences de la Vie et de la Terre, Ecole Normale Supérieure de Natitingou, Natitingou, Atacora, P.O. Box 72, Benin; 2 Agroecohealth Unit, International Institute of Tropical Agriculture, Abomy-Calavi, Atlantique, P.O. Box 0932, Benin; 3 Biorisk Management Facility, International Institute of Tropical Agriculture, Abomy-Calavi, Atlantique, P.O. Box 0932, Benin; 4 Ecole de Gestion et de Production Végétale et Semencière, Universite Nationale d'Agriculture, Ketou, Plateau, BP 43, Benin; 5 Laboratory of Cell Biology, Physiology and Immunology, Department of Biochemistry and Cellular Biology, Faculty of Sciences and Technology, University of Abomey-Calavi, Abomy-Calavi, Atlantique, 01 BP 526, Benin; 6 Laboratory of Experimental Physiology and Pharmacology, Faculty of Sciences and Technology, University of Abomey-Calavi, Abomy-Calavi, Atlantique, 01 BP 526, Benin

**Keywords:** Anopheles gambiae s.s., copper, malaria, tolerance, life traits, reproduction, Benin

## Abstract

**Background:**

*Anopheles* s.l. displays the potential to develop tolerance to heavy metals, particularly copper, this may occur at a significant biological cost, which can adversely affect its ecological fitness. This study investigated the larval metal exposure on larval development and reproduction of *An. gambiae* s.s., a laboratory susceptible strain, *kisumu.*

**Methods:**

Stage 2 larvae of *Anopheles gambiae*, *Kisumu* were exposed to C _1_ = 484 μg L ^-1^, C _2_ = 300 μg L ^-1^ and 0 μg L ^-1^ (control) of copper chloride. Larval mortality, pupation time, pupation rate, gonotrophic cycle length, fecundity and fertility of larvae/adults were assessed over six generations.

**Results:**

Results revealed that larval mortality rate was significantly higher in the C _1_ groups of each group (p = 0.000), but this mortality rate decreased over generations. Pupation time was extended to 13 and 14 days respectively for C _2_ and C _1_ groups (p = 0.000) compared to the control group. Similar results were observed for the gonotrophic cycle, which increased from 4 days at G0 to more than 6 days at generation 5 in adults of C _1_. The pupation rate in generation 4 (C _1_) and generation 5 of the same group (p = 0.000) as well as the emergence rate in generation 4 (C _2_, p = 0.000) and generation 5 (C _1_ and C _2_, p = 0.000) decreased significantly compared to the control group. The average number of eggs laid was lower in the test groups from generation 4 to generation 5 (C _1_ and C _2_, p = 0.00) and egg fertility was also negatively affected by exposure of the larval stage of *An. gambiae* s.s. to copper.

**Conclusion:**

This study showed that copper not only exhibits larvicidal properties in *Anopheles gambiae* s.s. larvae, it also revealed the potential of this metal to reduce fecundity and fertility in these malaria vectors.

## Plain Language Summary

The efficacy of insecticides used in vector control depends on the control of factors that contribute to the resistance of malaria vectors to these insecticides. Previous studies have reported the adaptation of *An. gambiae* s.l. larvae to breeding sites polluted by chemical contaminants. In this study, we pre-exposed 2 ^nd^ instar larvae of *Anopheles gambiae*, kisumu for 6 consecutive generations to 484 μg L ^-1^ (C _1_), 300 μg L ^-1^ (C _2_) and 0 μg L ^-1^ (control) until pupation. We found that after 3 days and 7 days, the mortality rate of larvae was a function of the concentration and was higher in the first generations before reaching zero at the end of the experiment. This pre-exposure negatively affected the pupation time, the pupation rate and the emergence rate. In adults, pre-exposure of larvae to copper lengthened the duration of oviposition after the blood meal and decreased the average number of eggs laid by females as well as the hatching rate compared to the control group. On the one hand, our study revealed the advantage of copper in vector control considering the larval mortality, the prolongation of the gonotrophic cycle and the pupation time that it induces and its negative action on fecundity and fertility on the other hand. However, the decrease in the mortality rate over the generations requires further work to elucidate the possibility of copper to select for resistance to insecticides.

## Introduction

The prevalence of mosquito-borne diseases is highly dependent on the ability of larvae to survive in breeding sites and on the ability of adult females to lay eggs in aquatic ecosystems ^1, 2^. The pollution of aquatic ecosystems due to the widespread and overuse of pesticides in agriculture is a cause for concern in vector control ^3^. Pesticide residues are therefore easily found in aquatic ecosystems through leaching, erosion and spray drift ^4^. Human activity has led to a large-scale increase in various environmental pollutants ^5, 6^. Except agriculture, industry, mining, household and municipal waste are the main sources of environmental pollution containing heavy metal ^7, 8^. Demographic growth in Africa, especially in urban areas, is increasing the load of these pollutants ^9^.

Copper is a metal naturally present in the environment through volcanic eruptions, plant decomposition, forest fires, marine aerosols and soil dust ^10^. This metal can easily find its way into the environment through discharges of industrial water, incineration of household waste, etc. and especially in the manufacture of agricultural fertilizers and pesticide (fungicides or algicides). According to Kabata-Pendias ^10^, copper is much more concentrated in light silty or clayey soils. In these small ecosystems, larvae of *An. gambiae* s.l., the main vector of malaria in Africa, are easily found. The presence of this vector in these polluted ecosystems represents a major change because it was known to shelter clear, sunny, unpolluted water ^11, 12^. According to these authors, the urbanization of cities in sub-Saharan Africa has enabled these vectors to adapt to xenobiotics.

Over the last decade, studies have focused on the impact of pesticides on the immature stage (larvae and pupae) and on the reproductive capacity of adult mosquitoes. These studies showed that atrazine and glyphosate influenced pupal emergence rate in *Aedes aegypti* and *Aedes albopictus* ^3^ as glyphosate and cypermethrin reduced the emergence rate of *An arabiensis* and *Culex quinquefasciatus* ^13^. Exposure of *Aedes aegypti* larvae to Roundup also reduced adults’ survival and their hatching time ^14^. Resistance of *An. gambiae* to deltamethrin following exposure of the larval stage to pesticides has recently been demonstrated ^15^.

Others studies have shown that non-pesticidal chemical residues modulate the capacity of detoxification enzymes ^16– 18^ as well as phenotypic resistance to insecticides ^19^. This regulation requires biological costs on the different developmental stages of the malaria vector and consequently on malaria transmission ^13, 16, 20^. Indeed, Copper plays an important role in insect immunity and in the regulation of proteins involved in melanization ^21, 22^. In *Ae. aegypti* and *An. gambiae* s.l., eggshell formation involves prophenoloxidases (PPO) and laccases (Lacs) ^23, 24^ which are all copper-dependent metalloproteins ^25^.

Recently, studies revealed that water, soil and even market garden produces are contaminated with lead, cadmium, chromium, zinc and copper in Benin ^26, 27^. In the absence of an effective management policy for solid and liquid, domestic and industrial waste, this heavy metal pollution of the various compartments of the biosphere cannot be reduced. Exposure of *An. gambiae* larvae to copper confers on its adult stages resistance to pyrethroids ^20^, insecticides mainly used in public health. However, vector transmission capacity depends on the insect's ability to survive and reproduce. In Benin, very little work has been done on *An. gambiae* s.s. life history vis a vis to metal pollution. This study aimed to understand the effects of copper on life traits of *An. gambiae* s.s., the major malaria vector in Benin.

## Methods

### Metal

Copper was used in the form of copper chloride dihydrate, CuCl2, 2H2O; purity 99.0% (Park Scientific Limited, 24 Low Farm Place, Moulton Park, Northampton, NN3 6HY, UK, Catalogue No. C11761). From this product, a stock solution of 1g L ^-1^ was prepared in a 35 mL bottle. Thus, 30.30 mg of copper chloride was dissolved in 30 mL of ultrapure water.The bottle was wrapped in aluminum foil then stored at 4°C for subsequent uses.

### Mosquito breeding

Susceptible strain of *An. gambiae* s.s., *kisumu* was reared in the insectary of the AgroEcoHealth platform of the International Institut of Tropical Agriculture (IITA-Benin). Mosquitoes (from eggs to adult stage) were reared under laboratory conditions (temperature 27°± 1°C, relative humidity 70–80% and a photoperiod of (12L: 12D). The larvae were fed with Tetramin baby (Tetra GmbH, Made in Germany, Herrenteich, Distributed in UK by Spectrum Brands, Manchester, Catalogue No. 879917) while the adults were fed with 10% sucrose (10g of local sugar diluted in 100 ml of distilled water) solution.

### Effect of copper on mortality of larvae, pupae and adults of *An. gambiae* s.s. *kisumu*

*Anopheles gambiae* s.s. larvae ( *kisumu* strain) were exposed to two different concentrations of copper chloride, the Lethal Concentration (LC _25_) (C _1_ = 484 μg L ^-1^), previously determined from nine concentrations of copper chloride (0 μg L ^-1^; 296.88 μg L ^-1^; 593.75 μg L ^-1^; 1187.5 μg L ^-1^; 2375 μg L ^-1^; 3166.67 μg L ^-1^; 4750 μg L ^-1^; 6333.33 μg L ^-1^ et 9500 μg L ^-1^) and a lower concentration (C _2_ = 300 μg L ^-1^) than this LC _25_. Nine (9) bowls of 4L capacity were placed on the shelf. Each concentration (C _1_, C _2_ and Control) was repeated three times (Three bowls per cpncentration) and bowls were filled with a final volume of two (2) liters. Distilled water was used as control group with no presence of copper. Three hundred (300) 2 ^nd^ instar larvae (G0) were transferred to each bowl (900 larvae for each concentration group) according to modified methods of Miah ^28^ who used third instar larvae. Furthermore, these larvae were exposed to these concentrations of copper throughout their larval stage. They were fed daily with Tetramin baby (Tetra GmbH, Made in Germany, Herrenteich, Distributed in UK by Spectrum Brands, Manchester). They were kept in insectary at 27±1˚C under 12:12 light: dark regime with 70–80% relative humidity and mortalities were recorded every morning. Pupae were counted daily in small cup and transferred to their respective cages covered with mosquito nets. Adults (G0) were fed with 10% sugar solution. The number of dead larvae, dead pupae and dead adults was recorded every day throughout the experiment and the larval mortality rate, mean of pupae recorded according to concentration were calculated in the control group as well as in the test groups. The pupation time was also determined as the day on which the maximum number of pupae was obtained ^5^. Thus, the adults obtained from each group were separated into two batches.

The first batch of adult mosquitoes (G0) was used to obtain the next generation (G1). Thus, the females (G0) of each group (Control, Concentration 1 and Concentration 2) were blood fed and allow to lay eggs in the petri dishes (Fisherbrand, USA) containing cotton soaked in distilled water and surmounted by a filter paper (Watman 3 mm/1 cm diameter). The eggs (G1) were placed in small bowls (250 ml) containing distilled water. After egg hatching, the larvae (G1) were transferred to large bowls according to the hatching rate of each group. These larvae (G1) were maintained until the 2 ^nd^ instar. At this stage, they were exposed to copper according to the procedure previously described at the same concentrations (484 μg L ^-1^, 300 μg L ^-1^). The monitoring remained the same until the adults were obtained (G1) and the cycle was repeated till generation 5.

### Determination of the gonotrophic cycle

In order to assess the effect of copper on reproduction, three parameters were recorded: the gonotrophic cycle, fecundity and fertility. The larvae of *An. gambiae, kisumu* were exposed to 484 μg L ^-1^ (C _1_) and 300 μg L ^-1^ (C _2_) as described previously. After emergence, 20 females and 40 males day-old mosquitoes from each treatment (concentration) were transferred to new cages ^20^. Each treatment was repeated three times (40 × 3 males and 20 × 3 females per Concentration), as well as the control. On the 3 ^rd^ day after emergence, the females were fasted for 6 hours and then blood-fed successively for three days ^29^. 48 h after the last blood meal, the 20 females in each cage were then introduced individually and gently into small cups containing cotton soaked in water and surmounted by a filter paper (Watman 3 mm/1 cm diameter). The cups were covered with mosquito net with cotton soaked in 10% sucrose was placed on the top of the net. Each cup was checked daily to identify females of *An. gambiae* s.s. that have laid eggs. After the first laying eggs mosquitoes were gently removed from the cup. Dead females before laying were also removed. They were transferred to a new eppendorf tubes containing cotton and silica gel and stored at -20°C for subsequent experiments. The time taken for the females to lay eggs was determined.

### Fecundity and fertility

To determine the fecundity and fertility, the number of mosquitoes that laid eggs and the number of eggs laid (fecundity) in each cup was counted under a binocular loupe (Wild 445111 Heerbrugg, Switzerland) every 24h ^29^.

The eggs from each mosquito were placed in 250 ml cup containing distilled water. The number of larvae hatched was counted every 24 hours for 96 hours to determine fertility. The experiment was carried out over 6 generations.

### Statistical analysis

All data collected were registered in Microsoft Excel 2016 software. The Kolmogorov-Smirnov test was used to assess the normality of the distribution. The chi-square test was used to compare larval mortality rates. Mean values of pupation time, pupation and emergence rate, gonotrophic cycle, eggs laid and eggs hatched were compared through the General Linear Model (GLM). In case the difference was reported Tukey's HSD test was used to identify these differences.

## Results

### The effect of larval exposure to copper on larval mortality

**Figure 1.**
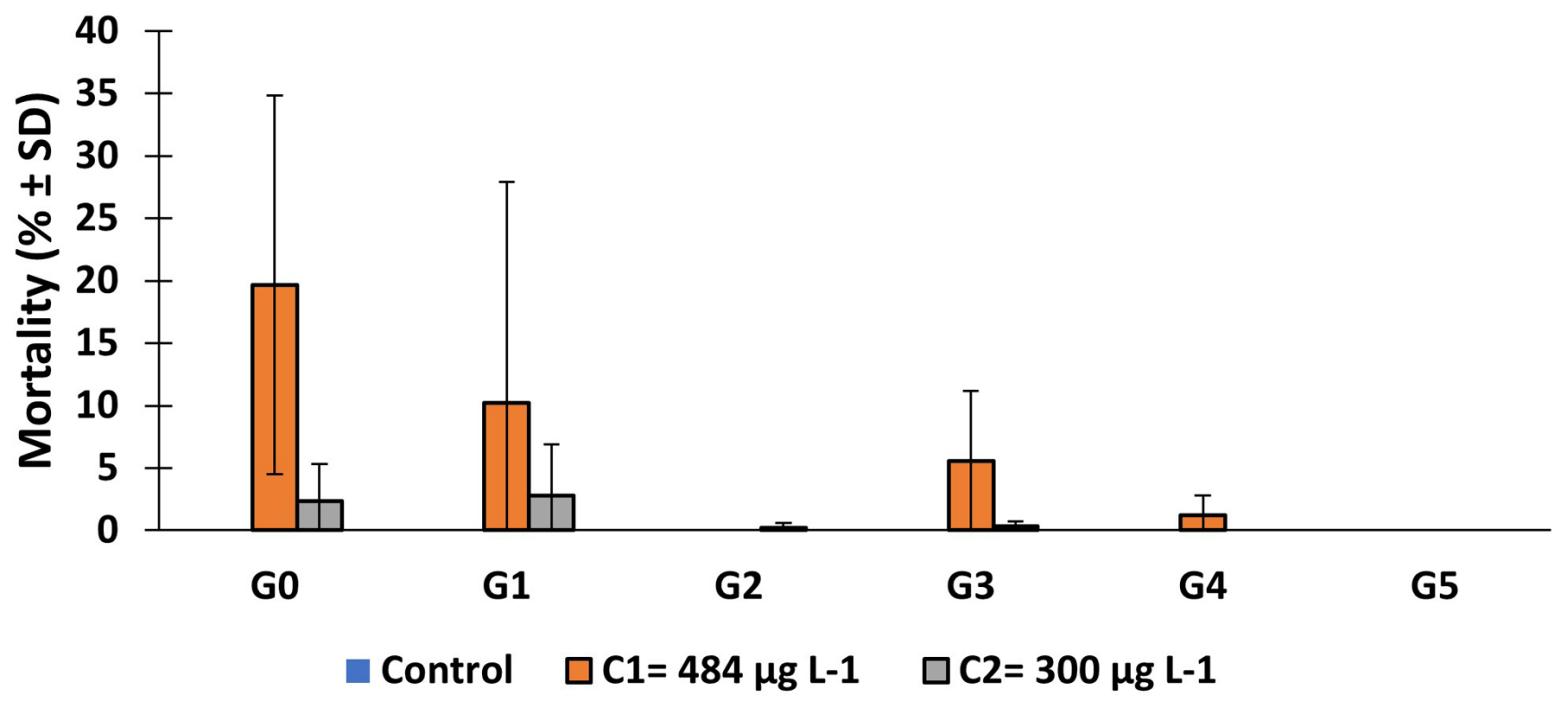
Mortality rate of larvae after 3 days of exposure to 484 μg L ^-1^ and 300 μg L ^-1^ copper. ‘G’ means Generation. SD means standard deviation.

The mortality rate of *An. gambiae* s.s., larvae after 3 days' exposure to copper varied according to concentrations ( Figure 1). For the same generation, the mortality rate increased according to the concentration. Except for generation 2 and the last generation (G5), mortality rate in test group (C _1_) increased significantly in all the generations compared to control group (generation 0: *X ^2^* = 305.79, *df* = 2, p = 0.000; generation 1: *X ^2^* = 121.39, *df* = 2, p = 0.000; generation 3: *X ^2^* = 94.28, *df* = 2, p = 0.000; generation 4: *X ^2^* = 22.09, *df* = 2, p = 0.0002). However, this rate decreased over the generations for C _1_ and C _2_ concentrations.

**Figure 2.**
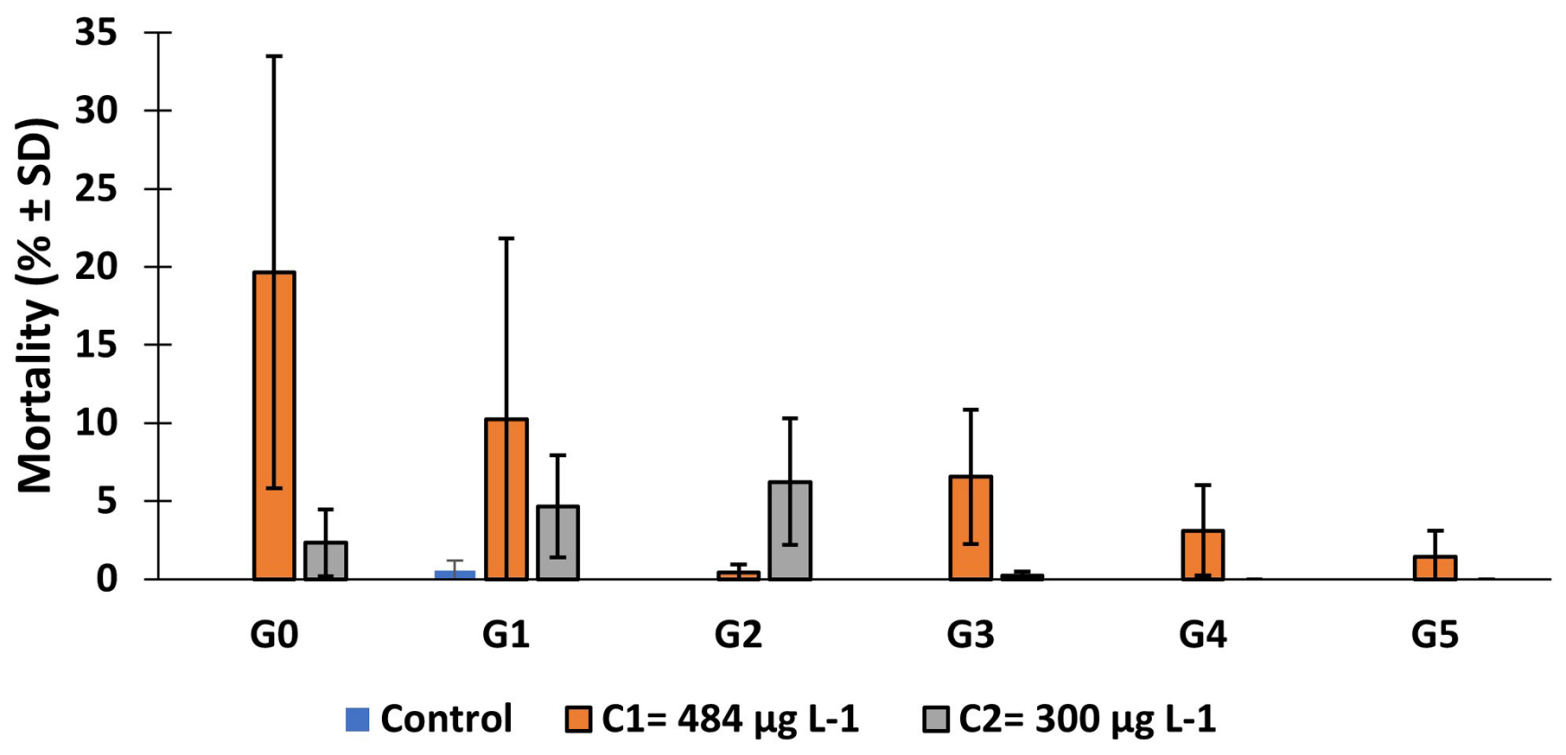
Mortality rate of larvae after 7 days of exposure to 484 μg L ^-1^ and 300 μg L ^-1^ copper. ‘G’ means generation. SD means standard deviation.

The mortality rate of *An. gambiae* s.s., larvae after 7 days' exposure to copper was calculated ( Figure 2). This mortality rate decreased from 19.67% in generation 0 to 1.44% in generation 5 for C _1_ while it was 2.33% and 0 in C _2_. In the first generation (G0), mortality rate in C _1_ group was significantly higher compared to control group ( *X ^2^* = 305.79, *df* = 2, p = 0.000). Similar results were observed in generation 1 ( *X ^2^* = 78.16, *df* = 2, 0.000), generation 2 ( *X ^2^* = 99.82, *df* = 2, 0.000), generation 3 ( *X ^2^* = 109.39, *df* = 2, p = 0.000), generation 4 ( *X ^2^* = 56.58, *df* = 2, p = 0.000) and generation 5 ( *X ^2^* = 16.05, *df* = 2, p = 0.0003) compared to control.

### The effect of larval exposure to copper on pupation time

**Figure 3.**
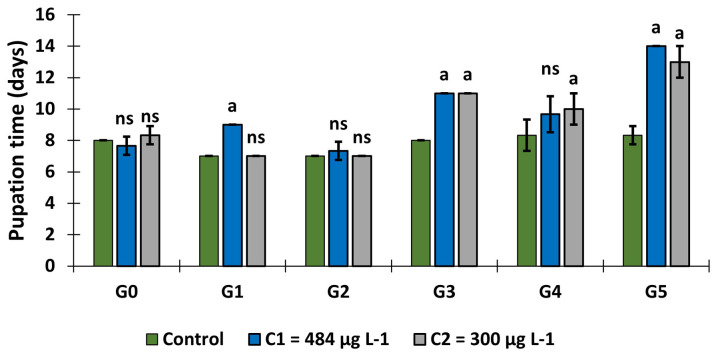
Variation in pupation time over generations following exposure of *An. gambiae* s.s. larvae to copper at C _1_ = 484 μg L ^-1^ and C _2_ = 300 μg L ^-1^. ‘G’ means Generation. ‘ns’ means “no significant difference with the control of the same generation”. ‘a’ means “significant difference with the control of the same generation” and ‘bars’ represent standard deviation. GLM: *F* = 16.55, *df* = 10, p = 0.000; n = 300 samples per each three repetitions.

In the control group, the average pupation time was 7.78±0.65 days over the 6 generations whereas it was 9.78 ± 2.46 days in C _1_ and 9.39 ± 2.39 days in C _2_ ( Figure 3). It was higher in the groups exposed to copper compared to control group (GLM: *F* = 16.55, *df* = 10, p = 0.000). In generation 1, pupation time increased in C _1_ group (9.00 ±0.00 days). This mean pupation time increased significantly in generation 3, with a fair value of 11.00±0.00 days in C _1_ and C _2_ and 10.00±1.00 days in C _2_ in generation 4. Finally, pupation time in generation 5 reached 14.00±0.00 days in C _1_ and 13.00±1.00 days in C _2_.

It should also be noted that this pupation time increased over the generations in the larvae exposed to copper ( *F* = 98.31, *df* = 5, p = 0.00), with a significant difference observed from generation 3 to the last generation.

### Effect of larval exposure to copper on pupation rates

The pupation rate was significantly lower in females only in (C _1_) group (GLM: F = 17.68; *df* = 10, p = 0.000), particulary in the generations 0, 4 and 5 (C _1_) compared to control group ( Table 1).

**Table 1.** Variation of average pupation rates ± standard deviation following exposure of larvae to copper. Values with asterisks indicate a significant difference on the same row. Different letters in the same column indicate a significant difference. GLM: *F* = 14.66, *df* = 10, p = 0.000; n = 300 for each three repetitions.

Generations	Control	C _1_ = 484 μg L ^-1^	C _2_ = 300 μg L ^-1^
**G0**	71 ± 16.70 ^b^	48.67±7.75 ^*b^	64.55±12.13 ^b^
**G1**	94.11±34.27 ^a^	96.33±15.71 ^a^	93.44±33.54 ^a^
**G2**	90.11±27.29 ^a^	98.33±23.01 ^a^	90.11±34.78 ^a^
**G3**	96.56±16.02 ^a^	91.22±10.27 ^a^	98.67±13.53 ^a^
**G4**	97.22±9.17 ^a^	67.56±6.84 ^*b^	80.22±9.73 ^b^
**G5**	96.22±12.46 ^a^	23.56±3.02 ^*c^	80.22±9.05 ^b^

### Effect of exposure of *An. gambiae* s.s., to copper on emergence rates

Exposure of *An. gambiae* s.s. larvae to copper (C _1_) significantly reduced pupal emergence rates (GLM: *F* = 14.66, *df* = 10, p = 0.000) in generations 4 and 5 while exposure to copper (C _2_) reduced emergence rate in generation 5 only. The results for emergence rates were summarized in Table 2.

**Table 2.** Changes in emergence rates (Average rate ± standard deviation) following exposure of larvae to copper over the generations. Values with asterisks indicate a significant difference on the same row. Different letters in the same column indicate a significant difference. GLM: *F* = 14.66, *df* = 10, p = 0.000 ; n = 300 for each three repetitions.

Generations	Control	C _1_ = 484 μg L ^-1^	C _2_ = 300 μg L ^-1^
**G0**	69.70±16.59 ^b^	67.42±12.42 ^b^	46.82±11.52 ^b^
**G1**	97.05±37.44 ^a^	98.04±16.47 ^a^	84.54±38.59 ^a^
**G2**	93.83±32.19 ^a^	98.08±23.72 ^a^	88.04±41.12 ^a^
**G3**	93.67±17.51 ^a^	93.17±11.79 ^a^	96.17±14.17 ^a^
**G4**	84.91±10.19 ^a^	69.08±10.75 ^*b^	89.47±12.78 ^a^
**G5**	95.73±13.17 ^a^	61.79±9.36 ^*b^	66.75±10.54 ^*a^

### Effect of copper on the gonotrophic cycle of *An. gambiae s.s.*

**Figure 4.**
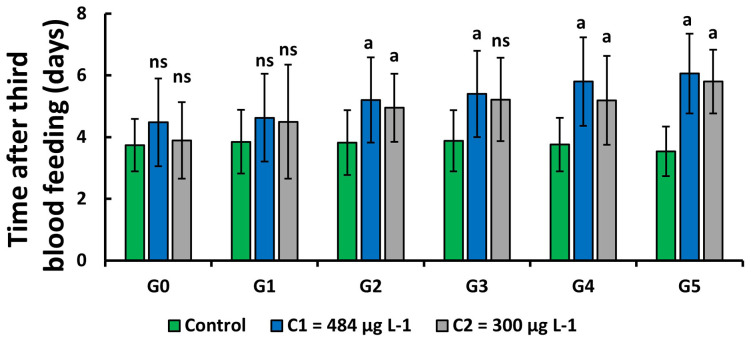
Effect of exposure of *An. gambiae*, kisumu larvae to copper on the number of days taken by females before laying eggs. ‘G’ means Generation. ‘ns’ means “no significant difference with the control of the same generation”. ‘a’ means “significant difference with the control of the same generation” and ‘bars’ represent standard deviation. Statistical analyses carried out on females laid among the 20 individual samples (GLM: *F* = 2.84, *df* = 9, p = 0.003).

The number of days taken by adult females to lay eggs after the last blood feeding was determined. For the two concentrations C _1_ and C _2_, mosquitoes took longer (4.48 days to 6.06 days in C _1_ and 3.89 days to 5.80 in C _2_ respectively from G0 to G5) to lay eggs than the control group (3.55 days to 3.88 days) ( Figure 4). This time increased slightly over generations in the test groups. It was significantly higher (GLM: *F* = 2.84, *df* = 9, p = 0.003) in C _1_ and C _2_ of the generation 2, generation 4 and generation 5 compared to control whereas it was only higher in C _1_ of the generation 3.

Larval exposure to 300 μg L ^-1^ of copper significantly increased the gonotrophic cycle over the generations ( *F* = 6.52, *df* = 4, p = 0.003), from generation 0 to generation 5. A significant increase was observed only between generation 0 and generation 5 (GLM: *F* = 6.59, *df* = 4, p = 0.004, Tukey test) on larvae exposed to 484 μg L ^-1^.

### Effect of copper on the fecundity of adult females *An. gambiae* ss.

**Figure 5.**
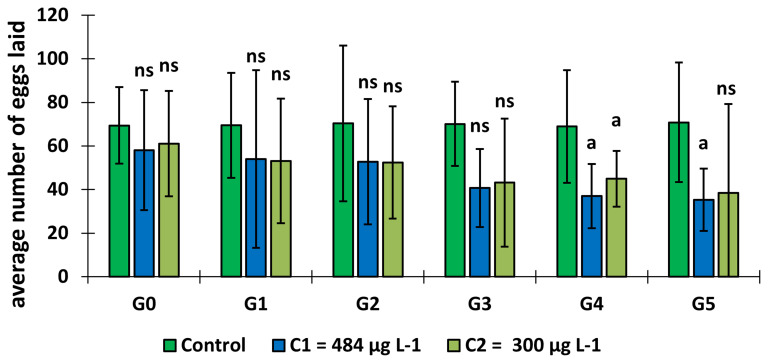
Variation in the mean number of eggs laid by female mosquitoes as a function of copper concentration. ‘G’ means Generation. ‘ns’ means “no significant difference with the control of the same generation”. ‘a’ means “significant difference with the control of the same generation” and ‘bars’ represent standard deviation. Statistical analyses carried out on females laid among the 20 individual samples (GLM: *F* = 1.87, *df* = 9, p = 0.05).

Mosquito larval exposure to copper had an effect on the fecundity of adults *An. gambiae* s.s. ( Figure 5). The average number of eggs laid per female decreased over the generations in the test groups and remained lower than in the control group (GLM: *F* = 1.87, *df* = 9, p = 0.05). Indeed, it was significantly lower in C _1_ and C _2_ at generation 4 and C _1_ at generation 5.

### Effect of copper on the adult fertility of *An. gambiae*, kisumu

**Figure 6.**
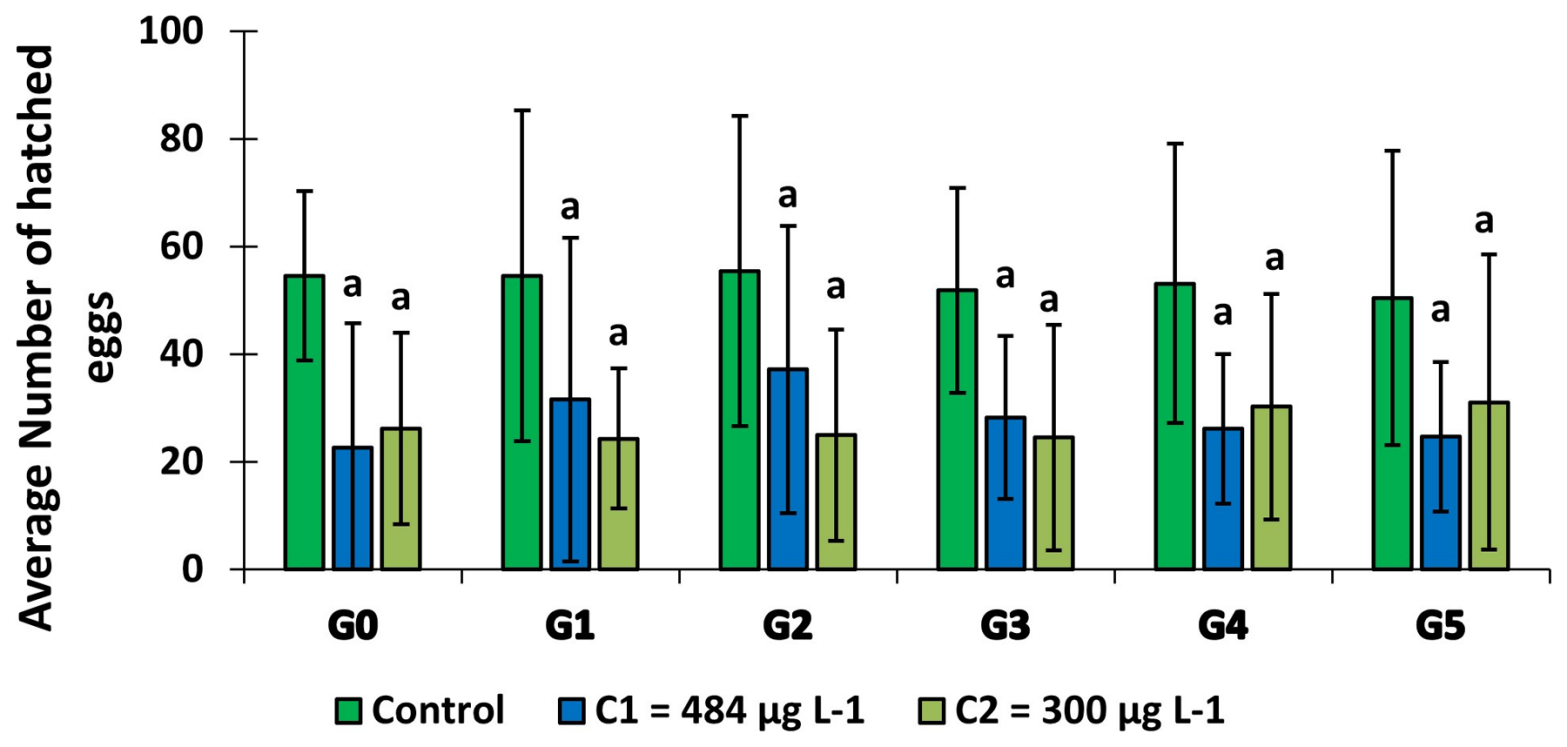
Average number of eggs hatched per female over the generations. G’ means Generation. ‘ns’ means “no significant difference with the control of the same generation”. ‘a’ means “significant difference with the control of the same generation” and ‘bars’ represent standard deviation.

The average number of eggs hatched in the groups which larvae were exposed to copper was much lower than in the control group ( Figure 6). In larvae exposed to 300 μg L ^-1^, this egg fertility was roughly equal over the first 4 generations (G0, G1, G2 and G3) before rising slightly in the last two generations. In contrast, in C _1 _ group, fertility increased from G0 to G2 and decreased from G2 to G5. For each generation, the fertility rate was significantly lower (GLM: *F* = 84,673, *df* = 2, p = 0.000) in the groups exposed to copper compared to the control group.

## Discussion

This study examined how frequent copper exposure affects the growth and reproductive capacity of *An. gambiae* s.s. The findings indicated that copper exposure led to increased mortality, extended pupation times, and negatively impacted the pupation rates of the larvae. Previous studies showed that 600 μg L ^-1^ and 1,18 μg L ^-1^ of copper adversely affects mosquito mortality and normal pupation durations ^20, 28^. Copper exposure has been linked to a decrease in the number of eggs produced by female mosquitoes. For example, when larvae were raised in environments with copper pollutants (1.86 μg L ^-1^ of copper nitrate which is the maximum acceptable toxic concentration legally allowed in the environment in South Africa), the resulting adults had significantly lower fecundity, which directly impacts population dynamics and vector control efforts ^20^. In this study, the observed larval mortality following the copper exposure highlights its toxicity, which disrupts various organ functions and vital processes within the insects. Copper exposure can alter the intestinal microbiota of larvae and pupae, which is crucial for digestion, nutrient absorption, and overall growth ^30^. This disruption likely contributes to the increased larval mortality seen in the experimental groups, as it results in significant energy loss necessary for survival. Additionally, copper presence in the environment induces stress in larvae, modifying their physiological responses, as noted in *Aedes aegypti* ^31– 33^. Exposure of *Aedes aegypti* larvae to 1.25 ppm copper induced a 58% reduction in lipids compared to mosquitoes raised in uncontaminated water ^31^. This concentration is below the standards recommended by the World Health Organization for water for human consumption (2000 μg L ^-1^) ^34^. This stress reduces lipid reserves during the transition to adulthood, diminishes body mass, and enhances starvation tolerance, effects that can extend to subsequent generations ^31^. Such adaptations to hunger in offspring may explain the reduction in larval mortality across generations observed in this study. Mosquito larvae appear to develop mechanisms to cope with heavy metal exposure like copper ^35^. This adaptation may facilitate the expansion of these vectors into polluted habitats, leading to heightened insecticide resistance ^5, 36^. Studies carried out in different ecosystems in Benin has shown the effective presence of copper in the sediments of the coastal lagoon at concentrations of up to 79,100 μg L ^-1^ ^37^ and in the composts used by market gardeners in Houeyiho at concentrations of 419 μg L ^-1^ ^38^. Results from this study revealed that copper exposure significantly impacts the pupation process in mosquitoes, particularly affecting the timing and success of this critical developmental stage as reported by Miranda and colleagues, showing the detrimental effects of copper on both pupation and emergence rates ^39^. The shift from stage 4 to the pupal stage, as well as from the pupal stage to adulthood, demands considerable energy reserves accumulated during the larval phase, since pupae do not feed. Copper disrupts the intestinal microbiota in larvae, leading to intestinal dysfunction due to altered microbiota, which hampers nutrient absorption and diminishes energy reserves necessary for molting, metamorphosis, and adult development ^40, 41^ as reported by numerous studies ^5, 42, 43^. Moreover, insects are characterized by their high reproductive capacity ^44^, hence, female mosquitoes require iron, obtained from blood meals, to lay eggs, but the eggs must also be viable for successful hatching ^45^. Copper negatively impacts the reproductive cells of mosquitoes, inducing apoptosis and adversely affecting egg viability ^20, 46^. In both *Aedes aegypti* and *An. gambiae* s.l., eggshell formation relies on copper-dependent metalloproteins, such as prophenoloxidases (PPO) and laccases (lacs) ^23– 25^. Additionally, copper exposure in mosquito larvae heightens oxidative stress, which detrimentally affects adult fecundity ^31^. The adverse effects on fertility and fecundity may stem from malformations in the midgut during post-embryonic development, impairing blood digestion. Females need a protein-rich blood meal to initiate vitellogenesis and produce eggs ^47^. Consequently, inadequate nutrient absorption or difficulties in digesting blood meals can result in non-viable eggs, thereby reducing fecundity and hatching rates ^48, 49^. Also, the differential expression of specific proteins, such as mucins, may account for the tolerance of *Anopheles* species to copper ^50^. Metal exposure can disrupt protein synthesis in mosquitoes, leading to the blockage of certain proteins and the overproduction of others ^50, 51^. Down-regulated proteins can impair various functions, including metabolic, immune, reproductive, and cellular growth processes ^51^. Conversely, some upregulated proteins might help mitigate the intracellular concentration of harmful substances ^50^. Copper alters the midgut epithelium of pupae, potentially compromising midgut function and mosquito survival. The gonotrophic cycle, which measures the frequency of blood meals, is crucial for assessing the potential for parasite acquisition and transmission ^52^. Extending the gonotrophic cycle is significant for vector control, as mosquitoes are known for their prolific reproduction ^25^. Mosquitoes can take multiple blood meals, resulting in numerous egg-laying events. A prolonged gonotrophic cycle is favorable for vector control, as it decreases the reproductive rate of these vectors, leading to a lower human bite rate and reduced malaria transmission ^29^. Overall, copper exposure adversely affects the pupation process in mosquitoes by prolonging pupation time and decreasing adult emergence rates. These findings underscore the potential of copper as a tool in mosquito control strategies, particularly when considering its effects on larval development and subsequent life stages.

## Conclusion

Exposure of *An. gambiae*, *kisumu* susceptible strain to copper over multiple generations led to a decrease in larval mortality, indicating that the larvae adapted to this metal. However, significant reductions were observed in pupation time, pupation rates, and the number of adults in the later generations, suggesting that this adaptation came at a cost to certain essential functions of the insect. The extended gonotrophic cycle, along with decreased fecundity and fertility, highlights the significant impact of copper on the reproductive capabilities of *An. gambiae* s.s. While previous studies have noted that copper can promote resistance to insecticides, it is important to recognize its potential benefits in vector control due to its adverse effects on the species' reproductive functions, which may help limit the spread of malaria vectors.

## Ethics and consent

Ethical approval and consent were not required

## Data Availability

Open Science Framework. Wellcome Trust: Exposure to copper metal enhances the tolerance of *An. gambiae* s.s. over multiple generations while reducing both fertility and fecundity in this primary malaria vector. https://doi.org/10.17605/OSF.IO/TFBM4 ^53^. **The project contains the following underlying data: ** Database for Reproduction parametters.xlsx Database for larval and pupae parametters.xlsx The data is available under the terms of the Creative Commons Zero “No rights reserved” data waiver (a CC0 1.0 Universal License)
