# Supplementary material for: Exposure to copper metal enhances the tolerance of An. gambiae s.s. over multiple generations while reducing both fertility and fecundity in this primary malaria vector
Source: Wellcome Open Res. 2025 Feb 5;9:623. Originally published 2024 Oct 28. [Version 2] doi: 10.12688/wellcomeopenres.23229.2 (PMC11803199; doi:10.12688/wellcomeopenres.23229.2)
Supplement: Peer review history

# Reviewer report: Reviewer response for version 1

**DOI:** 10.21956/wellcomeopenres.25585.r109849

**Published:** 2024-12-21

**Authors:** Isra Wahid

**Affiliations:** 1 Universitas Hasanuddin, Makassar, Indonesia

The manuscript addresses an important aspect of vector biology—the impact of heavy metal contamination, specifically copper, on the fitness of *Anopheles gambiae. *This study contributes to a growing body of data on anthropogenic pollutants, including copper as heavy metals, influence mosquito life history traits. The findings are significant as they explore the dual role of copper: as a stressor that negatively impacts mosquito fecundity and fertility and as a factor that promotes tolerance over generations. This duality has implications for vector control strategies and ecological management.

 The manuscript might be better if author add a deeper comparison of copper’s effects with other common pollutants to highlight its unique impact. While the research is novel in extending its focus across multiple generations, it did not explore into the molecular basis of the observed tolerance. It would strengthen the discussion section to include more recent studies on the molecular mechanisms of heavy metal-induced tolerance and resistance. which could bridge gaps in understanding the mechanisms underlying these adaptations.

 The experimental design is clear and includes appropriate controls. However, there are several methodological concerns: 1) The manuscript does not explicitly detail the number of larvae and adults used per replicate. A clear indication of sample sizes and consistency across generations would enhance the reliability of the findings. 2) The use of General Linear Models (GLM) and Tukey HSD tests is appropriate. However, the clarity of figures and legends could be improved by explicitly stating sample sizes, statistical tests used and p-values for all comparisons in the results and figures. 3) Also, assessing environmental copper levels in natural breeding habitats could strengthen the ecological relevance of the study by comparing laboratory concentrations to real-world conditions.

 The key findings were 1) initial increase in mortality followed by decreased mortality rates, indicating developing tolerance to copper; 2) significant delays in pupation and extended gonotrophic cycles, highlighting the biological costs of copper exposure; 3) decreased fecundity and fertility in copper-exposed groups, especially in later generations.

 The data are well-organized, but the interpretation could be improved by integrating broader ecological and evolutionary perspectives. For example, discussing how these findings align with known trade-offs in insect adaptation would provide a deeper understanding of the observed patterns. The discussion would be benefited by more detailed comparison with previous studies, particularly on other heavy metals and their impacts on mosquito biology, and by considering the implications of copper-induced tolerance for insecticide resistance, which is mentioned but not enough explored.

 The English used in the manuscript is generally clear but contains inconsistent use of terms like “Fo” and “G0.” 

 So, the recommendation for improvement is:

1. Clearly state sample sizes and replicate numbers in the methods section.
2. Improve figure legends to include statistical details and ensure clarity in presenting results.
3. Incorporate molecular analyses to identify mechanisms of copper tolerance in the discussion section, and discuss ecological implications more comprehensively, particularly in relation to vector control strategies.
4. Address inconsistencies in scientific terms used.

Is the work clearly and accurately presented and does it cite the current literature?

Partly

If applicable, is the statistical analysis and its interpretation appropriate?

Yes

Are all the source data underlying the results available to ensure full reproducibility?

Partly

Is the study design appropriate and is the work technically sound?

Yes

Are the conclusions drawn adequately supported by the results?

Yes

Are sufficient details of methods and analysis provided to allow replication by others?

Partly

Reviewer Expertise:

Mosquito ecology and behaviors, vector controls, and mosquito borne pathogen and diseases.

I confirm that I have read this submission and believe that I have an appropriate level of expertise to confirm that it is of an acceptable scientific standard.

# Reviewer report: Reviewer response for version 1

**DOI:** 10.21956/wellcomeopenres.25585.r111224

**Published:** 2024-12-09

**Authors:** Anjana Singha Naorem

**Affiliations:** 1 Cotton University, Guwahati, Assam, India

The present study conducted by Boukari and co-authors tests the various biological parameters post copper metal exposure to *Anopheles* *gambiae *larvae. the study is carried with two test concentrations, and they tried to compare the various important physiological changes post-treatment and found some interesting results. However, the findings are not well reported and represented. 

 Following are the key observations:

**Paragraph3:** Clarity in the introduction is lacking. When authors are citing the work of Bara *et al* 2014, Kibuthu *et al* 2017 it is not clear what kind of influence of the herbicides/pesticides/fertilizers they want to highlight on the mosquitoes through these studies.

 Spelling mistake of phenol oxidase and laccase in paragraph 4

**In the Methodology section,** in the third paragraph, I am apprehensive about the use of the term DOSE; The Term dose and concentration cannot be used interchangeably, both have different meanings. Authors have used both. My suggestion is to replace LD with LC and replace the term dose with concentration as we don’t know how much of the dose of the chemical has been absorbed or administered to the individual larvae. In the methodology section, it is unclear that out of 9 bowls, how many were used for each concentration and how many were control. Authors should specify it clearly that for each concentration how many bowls were chosen; there are two concentrations, and one control so did they use three bowls for each or 9 bowls for each concentration as well as the control? Also, mention the approximate number of 2 ^nd^ instar larvae in each bowl as it is important to understand the size of the sample. Mention that the mortality observation was taken on 3 ^rd^ day and on 7 ^th^ day post treatmemt.

 In the same paragraph, authors have used Fo for larvae and G0 for adult. The choice of the letters should be consistent. In the previous instance the letter F is followed by the letter ’o’ but later the letter G is followed by numeric ‘0’. Shouldn’t it be same as the numeric ‘0’?

 In the same paragraph, the line ““After emergence, the larval mortality rate, mean of pupae recorded according to concentration were calculated in the control group as well as in the test groups.”  is not clear and correct. I suggest correction.

  In the following two paragraph the correct the spelling of “Wattman” to “Whatman”.

 Under the “Determination of Gonotropic Cycle”, it is not clear in the second last line, when was the female removed and kept in silica gel- after the female died or after its first oviposition? Clarify that.

**Result section:**

 In the First paragraph, replace “excepted” with “except”.

 In Fig. 1 and 2, on Y axis mention Mortality (%) ±SD, rather than writing it in caption. Below the caption of every figure there should be mention of statistical analysis, P value, sample size, replications etc. There is a huge deviation seen in Fig. 1 and 2, this raises concern about the acceptability of the result or the claim that has been made by the author regarding the efficacy of Copper metal. Kindly include the sample size in each concentration every time, that will be able to explain this deviation

 Under pupation results, the average pupation time of Control is mentioned whereas avg pupation time of C1 and C2 concentrations are not mentioned. In the second line the concentration of C1 is mentioned, which is not needed, rather the result of pupation time of G1 should be mentioned clearly outside the paragraph and the value “9.00” should be followed by days.  The whole paragraph on pupation is very confusing and not written properly, this whole paragraph needs to be rewritten to show the result clearly. Moreover, in Fig. 3 on Y axis what is the unit ? isn’t the pupation time is in “days” next to pupation time. Below caption include all the details of the statistical analysis as said earlier.

 In both table 1 and 2 Title the sentence “Values with asterisks indicate a significant difference on the same line.” Here replace line with ‘row’ as line can mean horizontal as well as vertical means row or column. In Table 1 Authors forgot to mention that it is the ‘average rate’ shown in the table.

 I suggest authors to carefully repeat the statistical analysis as in fig. 4 there seem to be no significant treatments anywhere between the control and the treatments in any of the generations but they showed significant differences at G2, G3,G4 and G5. Y axis label is not proper and clear . Captions of both Fig 4 and 5 are same. How? Fig. 5 also clearly shows that no statistical difference exist anywhere in any generation, repeat the statistical analysis one more time. All SDs are very well overlapping with each other, how can there be any statistical difference?

 Grammatical error in the result of “Effect of larval exposure to copper on the adult fertility”. Rewrite it.

**Discussion** needs improvement. They should explain their every finding carefully. E.g. in larval mortality data after 3 days, why there is a very low mortality rate in C1 in 3 ^rd^ generation and then again increases. Moreover, in most of the result there is no significant difference was there between control and the treatments to highlight or to say categorically that the copper used in these concentrations are good enough to be considered for the mosquito control.  

 In the light of these observations, I suggest the authors to choose some more concentration and repeat the experiment or at least repeat the same experiment with more sample size and to do the statistical analysis carefully and report the result carefully. One more important point, authors failed to highlight clearly  the logic of using the given two concentrations C1 and C2? From whose study they found these LD50 and LD25. Moreover, it should be LC not LD. In the abstract, conclusion doesn’t clearly represent the findings that authors obtained in their experiment.

Is the work clearly and accurately presented and does it cite the current literature?

Partly

If applicable, is the statistical analysis and its interpretation appropriate?

Partly

Are all the source data underlying the results available to ensure full reproducibility?

Partly

Is the study design appropriate and is the work technically sound?

Partly

Are the conclusions drawn adequately supported by the results?

Partly

Are sufficient details of methods and analysis provided to allow replication by others?

Partly

Reviewer Expertise:

Insect control using botanical pesticides, Vector control, insect behaviour studies

I confirm that I have read this submission and believe that I have an appropriate level of expertise to confirm that it is of an acceptable scientific standard, however I have significant reservations, as outlined above.

# Reviewer report: Reviewer response for version 1

**DOI:** 10.21956/wellcomeopenres.25585.r108609

**Published:** 2024-11-19

**Authors:** Krystal Maya-Maldonado

**Affiliations:** 1 Johns Hopkins University, Baltimore, Maryland, USA

In this work, Gounou Boukari and co-authors explored copper exposure in *Anopheles gambiae.* The authors exposed larvae to different concentrations of copper chloride and evaluated several physiological effects over six generations. The introduction reads well and states the aim of the study. The work informs the field about the potential effects of copper exposure on mosquito life history traits over generations. There are interesting observations that need to be discussed. Moreover, the figures and results section need to be improved.  

**Figures:**

- Overall, the authors need to improve all the figure legends. Please verify that titles inform the reader according to the results. For example, the titles for Figure 4 and Figure 5 are the same; please verify the titles for each figure. Moreover, each figure legend must explain the statistical method used to analyze each data set, the number of individuals, or the sample size considered over the three biological replicates.
- The statistical analysis in Figures 4, 5, and 6 is confusing. The description of statistical analysis in material and methods indicated that the general linear model was used to determine differences, but the Tukey test was also used to identify possible differences. Describing the statistical method used in the figure legend would be preferable. This is important because Figures 4, 5, and 6 show a large standard deviation.
- **Figure 4. **What does the word “days” refer to in the y-axis? Are the authors referring to days after 3 ^rd^ blood feeding?

**Discussion section:**

- **Page 9. **The authors stated, “In this study, the observed larval mortality following the copper exposure highlights its toxicity, which disrupts various organ functions and vital processes within the insects”. It would be interesting to discuss copper concentrations used in previous reports and how close or far these concentrations are in the range of lethality for mosquitoes. Moreover, how similar are the concentrations used in this study compared to those in a natural environment where larvae are raised?
- According to Figure 4, the mosquitoes took more days after the second generation to start the gonotrophic cycle, suggesting a prolonged cycle. The last paragraph of the discussion pointed out the potential implications of extended gonotrophic cycles, but this fact is not compared with the results from Figure 4. In my opinion, there is interesting data that needs to be discussed more explicitly and compared with the literature.

**Minor comments:**

- **Page 4. Introduction section. **Paragraph #4. Please correct the misspelling in “Prophenoloxidases”, and the hyphen in the word “laccases” in the phrase “ *eggshell formation involves prophenoloxidas and lac-cases”.*
- **Page 4. Result section. “**Fo” and “G0” are used in the text. A consistent term needs to be used.
- **Page 9.** **Discussion section.** PPO and Lacs abbreviations can be mentioned before in the introduction.

Is the work clearly and accurately presented and does it cite the current literature?

Partly

If applicable, is the statistical analysis and its interpretation appropriate?

No

Are all the source data underlying the results available to ensure full reproducibility?

Yes

Is the study design appropriate and is the work technically sound?

Partly

Are the conclusions drawn adequately supported by the results?

Partly

Are sufficient details of methods and analysis provided to allow replication by others?

Partly

Reviewer Expertise:

Insect physiology, Insect immunity, metal ion homeostasis

I confirm that I have read this submission and believe that I have an appropriate level of expertise to confirm that it is of an acceptable scientific standard, however I have significant reservations, as outlined above.
